# Autoinflammatory disease: clinical perspectives and therapeutic strategies

**DOI:** 10.1186/s41232-022-00217-7

**Published:** 2022-12-02

**Authors:** Atsushi Kawakami, Endo Yushiro, Koga Tomohiro, Yoshiura Koh-ichiro, Migita Kiyoshi

**Affiliations:** 1 Department of Immunology and Rheumatology, Division of Advanced Preventive Medical Sciences, Nagasaki University Graduate School of Biomedical Sciences, Nagasaki, Japan; 2 Center for Bioinformatics and Molecular Medicine, Nagasaki University Graduate School of Biomedical Sciences, Nagasaki, Japan; 3 Department of Human Genetics, Division of Advanced Preventive Medical Sciences, Nagasaki University Graduate School of Biomedical Sciences, Nagasaki, Japan; 4 Department of Rheumatology, Fukushima Medical University School of Medicine, Fukushima, Japan

**Keywords:** Familial Mediterranean fever, *Mediterranean Fever* gene, Inflammasomes, Interleukin-1b, Interleukin-6, Interleukin-18

## Abstract

The molecular platforms of the innate immune system are essential to recognize pathologic external factors that are crucial to differentiate these danger signals from host motifs. A set of sensors recognizing pathologic factors is present and defined as a membrane-bound family of Toll-like receptors as well as the cytosolic ones including the family of nucleotide-binding domain leucine-rich repeat proteins. In this regard, the inflammasomes have been identified as an innate immune sensor toward pathologic external factors as well as endogenous damage-associated molecular pattern signals transducing from the above-mentioned receptors to gene expressions. Recent research has shown novel findings in inflammasome biology and genetics which lead to the alteration of diagnosis and management in autoinflammatory diseases as well as developing novel treatments, including the examples of nucleotide-binding domain leucine-rich repeat proteins-inflammasomes and pyrin-inflammasomes. The pyrin protein is encoded by the *Mediterranean Fever* gene on chromosome 16 that acts as a major regulatory component of the inflammasome, and is responsible for familial Mediterranean fever. We have recently examined the whole nucleotide sequence of the *Mediterranean Fever* gene in Japanese familial Mediterranean fever patients and revealed single nucleotide variants associated with the susceptibility of familial Mediterranean fever from a nation-wide survey by the next-generation sequencing. In a cytokine profile analysis of familial Mediterranean fever patients, we have found that interleukin-6 is considered to be one of the most crucial cytokines in familial Mediterranean fever attack since interleukin-6 had the best performance for distinguishing familial Mediterranean fever in attack from healthy controls or familial Mediterranean fever in remission, and in vitro interleukin-6 production is regulated by microRNAs-204-3p/phosphoinositide 3-kinase g pathway. Accordingly, we have been investigating the efficacy and safety of anti-human interleukin-6 receptor monoclonal antibody, tocilizumab, in patients with familial Mediterranean fever refractory or intolerant to colchicine through an investigator-initiated clinical trial supported by the Japan Agency for Medical Research and Development. Like interleukin-1b, interleukin-18 can be processed by caspase-1 and proteinase-3 to be activated within the inflammasomes. We have also found the importance of interleukin-18 in several autoinflammatory conditions. Recently, the concept of autoinflammation is widely distributed into many common diseases; thus, the attention to a wide spectrum of diseases *MEFV* gene deeply involved is required.

## Background

Inflammasomes are intracellular sensors that regulate host defense, cell homeostasis, and cell death. Upon activation, they recruit and activate caspase-1, which cleaves the proinflammatory cytokines pro-interleukin (IL)-1β, pro-IL-18, and gasdermin-D [1, 2]. Pyrin, a member of inflammasomes, is encoded by the *Mediterranean Fever (MEFV)* gene and contains PYD, bZIP (transcription domain), B-Box (zinc finger), CC (coiled-coil), and B30.2/SPRY domains. Thus, the *MEFV* gene, coding pyrin that acts as a major regulatory component of the inflammasome, is responsible for familial Mediterranean fever (FMF) [1, 2]. FMF is known as the most prevalent autoinflammatory disease characterized by some prominent clinical features of recurrent episodes of fever, serositis, arthralgia, and monoarticular arthritis [3]. Numerous variants have been identified in the region of *MEFV* in the INFEVERS database (https://infevers. umai-montpellier.fr/web/), and mutations in exon 10 reportedly correlate with disease severity and prognosis [3]. Accordingly, genetic diagnostic testing is considered to be important in the diagnosis and treatment of FMF, and FMF-causing pathogenic *MEFV* mutations favor an active pyrin state [1, 2]. In this regard, cytokine profiling and its expression regulatory mechanism have been investigated [4]. Clinical symptoms and signs of other rheumatic diseases as adult-onset Still’s disease (AOSD) are resemble with classical autoinflammatory diseases, and IL-1 and IL-18, inflammatory cytokines activated by a cytosolic activation platform of inflammasomes, are reported to be crucial. Thus, the understanding of inflammasome biology expands toward a wide range of inflammatory diseases. In this review, we summarize the recent advance of this field focusing on *MEFV* gene/FMF and related disorders.

## Main text

### MEFV gene and FMF

**Fig. 1:**
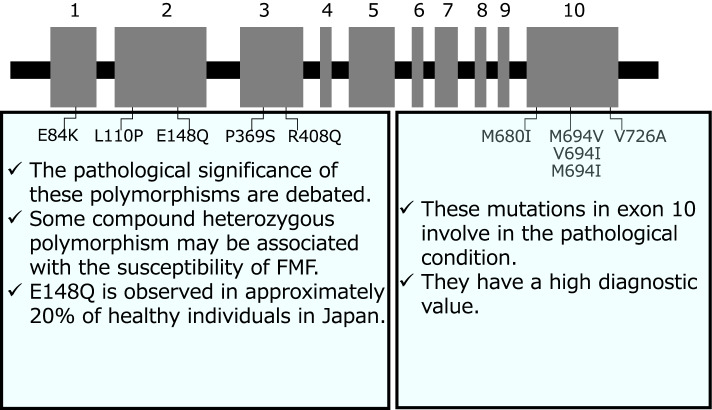
A domain structure of the *MEFV* gene. The *MEFV* gene is composed of 10 exons. The figure shows representative mutations and variants of the *MEFV* gene; however, according to the INFEVERS database (https://infevers. umai-montpellier.fr/web/), there are 389 sequence alterations that have been reported as of May 12, 2022

The pyrin protein (also known as marenostrin; TRIM20) is a 781-amino acid, ∼95-kDa protein encoded by *MEFV* on chromosome 16. Pyrin, five different domains are defined from homology search, is predominantly distributed to the innate immune lineage cells including granulocytes, eosinophils, monocytes, and dendritic cells. In particular, the C-terminal B30.2 domain of pyrin is considered to be functionally crucial since most of the FMF-associated mutations are concentrated in this domain that indicates an essential role of its domain regarding molecular pathways leading to clinical characteristics of FMF [3]. The causative gene for FMF, the *MEFV* gene, was found in 1997 through independent two investigator groups [2, 3], and FMF has long been considered as a recessive illness. The registry of Hereditary Auto-inflammatory Disorders Mutations, the INFEVERS database, has been established (see https://infevers. umai-montpellier.fr/web/) and a total of 389 mutations and variants of the *MEFV* gene is listed as of May 12, 2022. A domain structure of the *MEFV* gene including representative mutations and variants is shown in Fig. 1. The development of genetic testing of the *MEFV* gene in the clinical setting with the increment in diagnosed patients of FMF has reached the consensus that approximately 30% of the diagnosed FMF cases clinically carry only one demonstrable mutation despite the extensive search for a second disease-causing variant [2] which indicates that a single pathogenic *MEFV* mutation, with other genetic or environmental factors, might be sufficient to induce the activation of the pyrin inflammasome. FMF-associated missense mutations are thought to be present in exon 10 that encodes the B30.2/SPRY domain. In fact, an FMF mutation hot-spot within the B30.2/SPRY domain is distributed between amino acid residues 680 and 726 including Met680Ile, Met694Val, Met694Ile, and Val726Ala as the most frequent disease-causing mutations though either gain-of-function mutations or loss-of-function mutations in this region still debated. The carrier frequency is found as high as 10% in the Middle East and Mediterranean basin population, raising the possibility of balancing selection, but that in the Japanese population is low and a higher percentage of exons 2 or 3 variants in the *MEFV* gene compared with patients with FMF in Western countries [5]. In this regard, we have aimed to identify the whole nucleotide sequence of the *MEFV* gene in Japanese FMF patients and reveal single nucleotide variants (SNVs) associated with the susceptibility of FMF from a nation-wide survey by the next-generation sequencing (266 FMF patients and 288 controls) [6]. We identified the two most significant SNVs [rs28940578; M694I in exon 10, odds ratio (OR) = 153, *p* = 2.47×10^−21^ and rs3743930; E148Q in exon 2, OR = 1.65, *p* < 0.0005]. The stratified analysis identified rs28940578 as a risk allele in typical FMF. Haplotype AG, defined by rs401298 and rs28940578, was the most significant and prevalent among patients with typical FMF compared with controls (22.4% vs. 0%, respectively; OR = 137, *p* = 1.44×10^−31^). Haplotype GTC, defined by rs11466018, rs224231, and rs401877, was the most significant among patients with typical FMF without the rs28940578 mutation compared with controls (15.9% vs. 6%, respectively; OR = 12.4, *p* = 0.004). However, the GTC haplotype and the common missense variants in exons 2 and 3 are not decisive factors in the diagnosis of FMF [6], and the importance of clinical diagnosis should be emphasized. Accordingly, further investigation using more cases is required to determine the significance of this haplotype for the development of typical FMF. The DNA analysis of the whole *MEFV* revealed that typical cases without M694I and atypical cases were not caused by an abnormality on *MEFV* alone. Other factors including genetic factors may be involved in the susceptibility of FMF.

### Serum cytokine profile and FMF

As stated above in this review, IL-1 and IL-18 are crucial cytokines of FMF patients which also activate nuclear factor *k*B (NF-*k*B) signaling pathways that lead to increased amounts of NF-*k*B-mediated cytokines including tumor necrosis factor-a and IL-6 [4]. In line with these observations, it is widely known that elevated acute-phase proteins such as serum amyloid A and C-reactive protein and inflammatory cytokines are implicated in the disease activity of FMF in clinical practice. In this regard, we have analyzed the sera from FMF patients in attack or in remission by using a multisuspension cytokine array [7]. Under the investigation of 45 kinds of cytokine array, multivariate classification algorithms followed by a logistic regression analysis revealed that the combined measurement of IL-6, IL-18, and IL-17 distinguished FMF patients in attack from the controls with the highest accuracy (sensitivity 89.2%, specificity 100%, and accuracy 95.5%). Among the FMF patients, the combined measurement of IL-6, granulocyte colony-stimulating factor, IL-10, and IL-12p40 discriminated febrile attack periods from remission periods with the highest accuracy (sensitivity 75.0%, specificity 87.9%, and accuracy 84.0%). These findings help to improve the diagnostic performance of FMF in daily practice and extend our understanding of the activation of the inflammasome leading to enhanced cytokine networks. We also found that serum IL-18 in remission was significantly higher in the FMF patients with *MEFV* exon 10 mutation than in those without mutation [7], and furthermore, our recent study showed that the coexistence of *MEFV* exon 2 variants boosted serum IL-18 levels in remission among patients with FMF that had a heterozygous *MEFV* exon 10 mutation [8]. Therefore, considering these cytokines, it is suggested that the coexistence of *MEFV* exon 2 variants may have additional effects on more severe phenotypes and subclinical inflammation in patients with FMF with *MEFV* exon 10 mutation.

MicroRNAs (miRNAs) are endogenous small RNAs that post-transcriptionally regulate gene expression by pairing with a target [9]. A number of circulating miRNAs associated with inflammation have been identified [9], and our study found that miR-204-3p was the most important miRNA, the most down-regulated miRNAs, during FMF attack [10]. miR-204-3p regulates the production of toll-like receptor (TLR) 4-related inflammatory cytokines by targeting 3^0^-UTR regions of PIK3CG. These data thus suggest that miR-204-3p serves as a suppressor of inflammatory cytokine production in FMF by targeting the phosphoinositide 3-kinase (PI3K) g pathway. IL-6 is considered to be one of the most crucial cytokines in FMF attack since IL-6 had the best performance for distinguishing FMF in attack from healthy controls or FMF in remission [7], and in vitro IL-6 production is regulated by miR-204-3p/ PI3Kg pathway [10]. Accordingly, we have been investing the efficacy and safety of anti-human IL-6 receptor monoclonal antibody, tocilizumab, in patients with FMF refractory or intolerant to colchicine through investigator-initiated clinical trial supported by Japan Agency for Medical Research and Development, AMED [11, 12].

### IL-18 and inflammatory diseases

In comparison to IL-1β, another IL-1 family member cytokine of IL-18 has been less studied. Like IL-1β, IL-18 is reported to be processed by caspases-1/3 that forms the mature 18 (activated form) [4]. Therefore, IL-18 could also be involved in human inflammatory diseases as IL-1β [4]. IL-1β is biologically active within the pg/mL range; however, the biologically active concentrations of IL-18 are said to be around 10–20 ng/mL and sometimes higher levels are necessary in case of the activation in vitro [4]. Ubiquitous tissue expression of the IL-18 precursor is found; thus, IL-18 signaling could be manipulated by concentration-dependent manner [4]. The delivery of low affinity between mature IL-18 and IL-18 receptor alpha chain (IL-18Rα) is formed. However, in case an IL-18 receptor β chain (IL-18Rβ) is also expressed on the cells, a high-affinity ligand-receptor complex is developed like the IL-1R accessory chain IL-1R3. The heterodimer mature IL-18 ligand-receptor complex recruits MyD88 through the Toll-IL-1 receptor (TIR), four IRAKs, and TRAF-6 which leads to the activation of NF-κB, similar to IL-1 signaling [4].

Serum IL-1β concentration in general is very low compared with IL-18 [4]. In this regard, we did not find a significant difference in sera concentration of IL-1β among FMF in attack, FMF in remission, and healthy controls whereas sera IL-18 concentration of patients of FMF in attack was much higher compared with FMF in remission or healthy controls [7]. Comparing sera concentration of IL-18 in FMF in attack with that in other inflammatory diseases as AOSD [13], that of IL-18 in the active phase of AOSD is extremely high [14]. As mentioned earlier, IL-18 is initially synthesized as an inactive precursor and the cleavage for processing into a mature, active molecule is mediated by pro-inflammatory caspases following the activation of inflammasomes [4]. In this regard, we have developed several monoclonal antibodies which react specifically with mature IL-18 processed by caspases [14]. We have found that most of serum IL-18 in the active phase of AOSD attributes to mature IL-18 [14]. The literature has found that other autoinflammatory/autoimmune diseases including systemic lupus erythematosus (SLE), rheumatoid arthritis (RA), type-1 diabetes mellitus, Crohn’s disease, psoriasis, and graft versus host disease are thought to be mediated by IL-18 [4]. In addition, we have recently found the participation of *MEFV* gene variants (exon 2 or 3), not exon 10 mutations, in patients with idiopathic multicentric Castleman disease (iMCD) [15], which might associate with cytokine/chemokine storm found in iMCD [16].

## Further directions and conclusions

Inflammasomes are crucial sensors toward external pathogens and endogenous damage-associated molecular pattern signals. Tissue-specific expression as well as the differences of those triggers are some considered to be explanatory reasons for some phenotypic differences in autoinflammatory diseases. The *MEFV* gene is considered to contribute essentially in the activation of inflammasomes resulting in the activation of inflammation. An understanding of autoinflammatory mechanisms/diseases would encompass across the immunological disease spectrum, and it has been realized that several tissue-specific factors could contribute to inflammation [17]. Based upon this hypothesis, common diseases with evident inflammatory parameters could be classified into predominantly autoinflammatory in nature, although most of these diseases also have signs of autoimmunity [17]. It is considered to be important that this way of thinking of autoinflammation may lead to the establishment of boundaries/borderlines for what constitutes autoinflammatory reactions. Considering nucleotide-binding domain leucine-rich repeat (NLR) proteins 3 (NLRP3), NLRP3 inflammasome activation and IL-1β/IL-18 secretion have recently emerged as a central mechanism in the pathogenesis of diseases associated with NLRP3 activation by danger signals like gout, pseudogout, Alzheimer’s disease, or type 2 diabetes [18]. *MEFV* gene mutation modulates the NLRP3 activation [1, 2]. These observations strongly suggest the importance in concept of autoinflammation toward a wide spectrum of diseases *MEFV* gene deeply involved in these processes.

## Data Availability

Not applicable.

## Abbreviations

AOSD: Adult-onset Still’s disease
IL: Interleukin
FMF: Familial Mediterranean fever
*MEFV* gene: *Mediterranean Fever* gene
miRNAs: MicroRNAs
iMCD: Multicentric Castleman disease
NF-*k*B: Nuclear factor *k*B
NLR: Nucleotide-binding domain leucine-rich repeat
PI3K: Phosphoinositide 3-kinase
