# Correction: Autoinflammatory disease: clinical perspectives and therapeutic strategies

**DOI:** 10.1186/s41232-022-00251-5

**Published:** 2022-12-29

**Authors:** Atsushi Kawakami, Yushiro Endo, Tomohiro Koga, Koh-Ichiro Yoshiura, Kiyoshi Migita

**Affiliations:** 1 Department of Immunology and Rheumatology, Division of Advanced Pre- ventive Medical Sciences, Nagasaki University Graduate School of Biomedical Sciences, Nagasaki, Japan; 2 Center for Bioinformatics and Molecular Medicine, Nagasaki University Graduate School of Biomedical Sciences, Nagasaki, Japan; 3 Department of Human Genetics, Division of Advanced Preventive Medical Sciences, Nagasaki University Graduate School of Biomedical Sciences, Naga- saki, Japan; 4 Department of Rheumatology, Fukushima Medical University School of Medicine, Fukushima, Japan

**Correction: Inflamm Regen 42, 37 (2022)**

**https://doi.org/10.1186/s41232-022-00217-7**

Following publication of the original article [1], authors found that the first and last names of the authors (except the first author) were appearing reversed.

The original version was:

Atsushi Kawakami^1*^, Endo Yushiro^1^, Koga Tomohiro^1,2^, Yoshiura Koh-ichiro^3^ and Migita Kiyoshi^4^

The correct authorship has been updated in this Correction.

The original article [1] has been corrected.
